# Metastasis of ovarian cancer is mediated by kallikrein related peptidases

**DOI:** 10.1007/s10585-013-9615-4

**Published:** 2013-09-17

**Authors:** Ying Dong, Daniela Loessner, Helen Irving-Rodgers, Andreas Obermair, James L. Nicklin, Judith A. Clements

**Affiliations:** 1 Cancer Program, Institute of Health and Biomedical Innovation, Queensland University of Technology, 60 Musk Avenue, Kelvin Grove, Brisbane, QLD 4059 Australia; 2 Translational Research Institute, 37 Kent Street, Wooloongabba, QLD 4102 Australia; 3 Present Address: School of Medical Science, Griffith University, Gold Coast Campus, Southport, QLD 4222 Australia; 4 Department of Gynaecological Oncology, Royal Women’s Hospital, Herston, QLD 4101 Australia; 5 Department of Gynaecological Oncology, Wesley Research Institute, Auchenflower, QLD 4066 Australia

**Keywords:** Kallikrein-related peptidases, Serous ovarian cancer, Ascites microenvironment, Multicellular aggregation, Chemoresistance, Metastasis

## Abstract

Ovarian cancer, in particular epithelial ovarian cancer (EOC), is commonly diagnosed when the tumor has metastasized into the abdominal cavity with an accumulation of ascites fluid. Combining histopathology and genetic variations, EOC can be sub-grouped into Type-I and Type-II tumors, of which the latter are more aggressive and metastatic. Metastasis and chemoresistance are the key events associated with the tumor microenvironment that lead to a poor patient outcome. Kallikrein-related peptidases (KLKs) are aberrantly expressed in EOC, in particular, in the more metastatic Type-II tumors. KLKs are a family of 15 serine proteases that are expressed in diverse human tissues and involved in various patho-physiological processes. As extracellular enzymes, KLKs function in the hydrolysis of growth factors, proteases, cell membrane bound receptors, adhesion proteins, and cytokines initiating intracellular signaling pathways and their downstream events. High KLK levels are differentially associated with the prognosis of ovarian cancer patients, suggesting that they not only have application as biomarkers but also function in disease progression, and therefore are potential therapeutic targets. Recent studies have demonstrated the function of these proteases in promoting and/or suppressing the invasive behavior of ovarian cancer cells in metastasis in vitro and in vivo. Both conventional cell culture methods and three-dimensional platforms have been applied to mimic the ovarian cancer microenvironment of patients, such as the solid stromal matrix and ascites fluid. Here we summarize published studies to provide an overview of our understanding of the role of KLKs in EOC, and to lay the foundation for future research directions.

## Introduction

Ovarian cancer represents a growing women’s health issue worldwide [1–3]. In Australia, the risk of a female being diagnosed with ovarian cancer is 1 in 79 [1]. While the age-standardized incidence rate of ovarian cancer is decreasing, the actual number of women diagnosed with ovarian cancer in Australia is increasing and is projected to increase further due to the ageing of the population. In the US, 22,240 new cases will be diagnosed and 14,030 women will die from this cancer in 2013 [3]. The majority of ovarian cancers are classified as epithelial ovarian cancers (EOC, ~90 %) with the patients’ mean age at 62 years. Given the lack of early warning signs and the lack of effective screening, three quarters of patients with epithelial ovarian cancer (EOC) are diagnosed at advanced stages 3 or 4, thus implying spread throughout the peritoneal lining, parenchyma of the liver or pleura [4]. Treatment comprises a combination of aggressive surgery plus cytotoxic chemotherapy [5]. Morbidity of treatment is considerable and prognosis remains typically poor worldwide [1–3]. While most patients respond to chemotherapy initially, the tumor eventually becomes chemo-resistant which leads to relapse [6], with an average disease progression free survival of 18 months [7] and a less than 30 % 5-year overall survival rate [8]. By contrast, survival outcomes are generally good for women diagnosed with non-epithelial ovarian cancer or with EOC incidentally found at an early stage.

In the past three decades, our understanding of EOC has been extended into its origin, classification and genetic features. Studies on the characterization of metastasis of ovarian cancer have led to the search for new biological agents as part of therapeutic regimens. It is well known that metastasis is associated with the invasive behaviors of tumor cells in which cell membrane proteins, receptors and extracellular matrix (ECM) proteins play important roles [6]. As in other cancer types, cancer-associated proteases are a crucial component in the regulation of these proteins via hydrolysis, such as matrix metalloproteinase (MMP)2, MMP7, MMP9 and urokinase plasminogen activator (uPA) [9–11]. Recent studies have revealed that most kallikrein-related peptidases (KLKs), a family of serine proteases, are aberrantly expressed in cultured ovarian cancer cells and patient specimens and may also play a role in cancer progression and in particular metastasis [12, 13]. In the following sections, we will focus on the pathogenesis of EOC, the expression and function of KLKs in EOC, and the challenges faced in the treatment of this cancer.

## Pathogenesis of EOC

The normal ovary is covered by a single layer of flat or cuboidal mesothelial cells. It has been hypothesized that EOCs originate from these ovarian surface mesothelial cells. EOC has been recognized as a heterogeneous and complex disease with predominant serous, mucinous, clear cell, endometrial and transitional cell types. However, recent studies have revealed more genetic and molecular phenotypes that help us better understand the clinical features as listed below.

Challenging the conventional hypothesis, recent pathological studies have proposed that ovarian cancer might in fact generate from seeding of cancer cells from the fallopian tube to the ovary [14–17]. With combined analysis of clinical, histopathological and genetic characteristics, Kurman and colleagues [17, 18] have proposed a two-pathway model of this tumor. In particular, they accounted for the difference of mutation frequencies of *KRAS*, *BRAF* and *TP53* dividing EOC into two categories, Type-I and Type-II tumors. Type-I tumors include serous, endometrioid, mucinous, and clear cell types, but display low-grade nuclear and architectural features, slow growth, and can be associated with well-defined benign and borderline (low malignant potential) ovarian precursor lesions. Most often genetic variations of Type-I tumors are *KRAS* and *BRAF* mutations that activate the mitogen-activated protein kinase (MAPK) signaling pathway [19–21]. Additionally, Type-I tumors are more frequently found with mutations of *ARID1A*, *ERBB2*, *PTEN*, *CTNNB1* and *PIKCA* [22]. On the other hand, these alterations are rarely seen in Type-II tumors which are frequently (>80 % of cases) detected with mutations of *TP53*, *CCNE1* and chromosome instability [17, 22]. Type-II tumors are rarely linked to benign or borderline ovarian precursor lesions, but comprise almost all of high grade serous EOC, with clinical features of a high growth rate, metastasis and a less than 30 % 5-year patient survival rate [23]. Using integrated genomic analyses, these Type-II tumours and in particular the high grade serous EOCs are subdivided into differentiated, immunoreactive, mesenchymal and proliferative groups due to their different gene signatures [24].

Relevant to ovarian cancer metastasis, and critically due to the lack of an anatomical barrier around the ovary and exposure to the peritoneal space, individual EOC cells or small cell clusters are shed from the primary tumor into the abdominal cavity. These tumor cells adhere to and disrupt the peritoneal mesothelial cells, exposing the Type-I/III collagen-rich ECM underneath the peritoneal membrane. The EOC cells adhere strongly to the exposed ECM, in comparison to the mesothelial cells which adhere more strongly to the interstitial Type-I collagen [25]. Another clinical feature indicative of a poor prognosis for patients with this malignancy, in particular high grade serous EOC, is the formation of ascites fluid [26]. More than 70 % of EOC patients, in particular those with Type-II tumors, present with a pool of fluid in their abdominal cavity (ascites/effusions) harboring a population of tumor cells [26]. This is due to obstruction of lymphatic vessels by implanted tumor, preventing the outflow of fluid that leaks from disorganized tumor vessels and thus accumulating ascites [27]. In this scenario, patients with the aggressive Type-II tumors, in particular high grade serous EOCs, not only have metastatic tumors that grow in solid stromal matrices but also a cell population suspended in the ascites fluid [4, 6, 28]. Similar to the tumor cells in the solid matrices of the metastatic sites, the EOC cells derived from ascites fluid are resistant to various therapeutic regimes [28]. In particular, a recent study demonstrated that a subpopulation of EOC cells expressing CA125, EpCAM and STAT3, is able to grow as three dimensional (3D) suspension spheroids or multicellular aggregates (MCAs) that are tumorigenic and resistant to chemo-treatment [29]. These CA125, EpCAM and STAT3 positive ascitic EOC cells are the source of peritoneal adhesions leading to a poor outcome of women diagnosed with this disease. However, the mechanisms underlying metastasis to the peritoneal membrane remain to be elucidated.

## Kallikrein-related peptidases (KLKs)

**Fig. 1:**
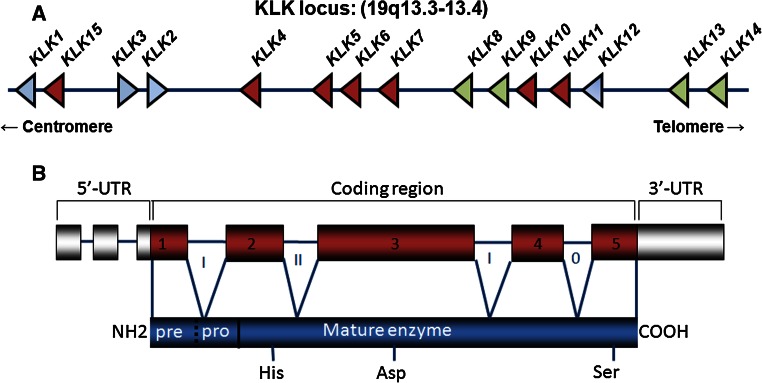
Genes and exon/intron organization of kallikrein-related peptidases. **a** Location of the KLK locus at chromosome 19q13.3-13.4. Schematic representation of the interval between D19S425 and D193418. The KLK locus is located proximal to D19S418. The position of the 15 kallikrein encoding genes on the KLK locus is marked. KLK1, KLK15, to KLK14 are transcribed telomere to centromere, whereas KLK2 and KLK3 are transcribed in the opposite direction. KLKs highlighted in *dark* *red* are representative of their aberrant expression associated with poor outcome for ovarian cancer patients, while *green* indicates an association with favorable patient outcome. **b** Schematic showing the organization of 5′-untranslated region (5′-UTR), coding region (*exons* and *introns*), and 3′-UTR of KLKs (*top panel*) and KLK protein structure (*bottom panel*). Catalytic triad, His, Asp and Ser, as indicated

The kallikrein locus, with 15 members in this family, spans approximately 265 kb on chromosome 19q13.3-13.4 forming the largest continuous cluster of human proteases (Fig. 1a) [30–34]. Each KLK gene contains 5′ and 3′-untranslated region, 4 introns and 5 exons encoding each of the KLKs (Fig. 1a, b). The KLKs are synthesized as pre-pro-peptidases, with the pre-signal peptide crucial for secretion of these enzymes (Fig. 1b). Cleavage of the pro-peptide is required for activation to the mature enzyme with appropriate conformation of the catalytic triad, histidine (His), aspartate (Asp) and serine (Ser). Members of the KLK family have trypsin or chymotrypsin-like substrate specificity. KLK1, KLK2, KLK4–KLK6, and KLK10–KLK15 have trypsin-like specificity for cleavage after argine and lysine residues [35, 36]. KLK15 also has trypsin-like specificity but cleaves after a glutamate residue [37]. On the other hand, KLK3 and KLK7 display chymotrypsin-like specificity for tyrosine, leucine, and phenylalanine residues [13, 38]. Given this specificity and that most KLKs require an enzyme with trypsin-like specificity for activation, it is not surprising that KLK activation cascades can occur in many tissues [13, 39, 40]. It has also been documented that KLKs can be activated by other proteases with a recent biochemical study showing that matrix MMP20 cleaves pro-KLK1–4, pro-KLK6, pro-KLK7, pro-KLK9, pro-KLK11 and pro-KLK15 [41].

Like other serine proteases, once KLKs are activated, their proteolytic action is irreversible and controlled by endogenous inhibitors [42]. The endogenous inhibitors targeting distinct KLKs have been identified, such as zinc ions, Kazal-type inhibitors and α2-microglobulin [43, 44]. Recently, the naturally occurring 14 residue long cyclic sunflower trypsin inhibitor (SFTI of the Bowman-Birk family) was redesigned to inhibit specific kallikreins using a combination of molecular modeling and matrix substrate screening [45]. SFTI–FCQR selectively blocks the proteolytic activity of KLK4 and reduces the activity of the proteinase activator receptor (PAR)2, a KLK4 substrate, in a cell-based assay [45]. In addition, the SFTI–FCQR Asn14 variant can also inhibit KLK4, and importantly, this variant displayed bioactivity in an in vivo mouse model [46]. Using a similar approach, SFTI–WCTF was selected to potently inhibit KLK7 proteolytic activity at a nanomolar concentration [47].

From a clinically relevant aspect, tissue-specific association of KLKs has been identified. For instance, KLK2, prostate-specific antigen (PSA)/KLK3, KLK4 and KLK11 are expressed at higher levels in prostate tissue extracts and seminal fluid, KLK5–11 in skin, and KLK6 and KLK8 in cerebrospinal fluid [48] where the activation cascades noted above can occur. It has become evident that expression of many KLKs is regulated by steroid hormones in a tissue-specific manner, such as KLK2, PSA/KLK3 and KLK4 by androgen in prostate and breast cancer cells, and KLK4 by estrogen in endometrial and ovarian cancer cells [49–52].

## Patho-physiological roles of KLKs

It has been well documented that KLK enzymes have extracellular hydrolysis activities, such as activating and/or degrading their substrates, including growth factors, ECM proteins, other cancer-associated proteases, cell membrane bound receptors and adhesion proteins. Just as KLK family members have a diverse tissue/organ expression profile, they also have a wide range of physiological functions. For example, KLK1 has a role in the cardiovascular system via cleavage of the low molecular weight kininogen to generate bradykinin, which stimulates its receptor, the bradykinin receptor 2 [53]. KLK4 is involved in the remodeling of the organic matrix and disruption of intercellular junctions in tooth development via hydrolysis of enamelin [54, 55] and amelogenin [56]. KLK4 can also activate pro-meprin β and may have a role in keratinocyte migration in skin [57]. In addition to KLK4, a recent study has identified that KLK5 and KLK8 cleave meprins, suggesting their role in this process, and interestingly, KLK5 can also cleave the other subunit of the family, pro-meprin α [58]. KLK5 and KLK7 are involved in keratinization, stratum corneum formation, turnover and desquamation of the skin, via degradation of the cell adhesion glycoproteins, corneodesmosin and plakoglobin [59–61]. These two enzymes have also been localized to numerous other tissues, and their function via hydrolysis to degrade cell membrane bound adhesion glycoproteins, has been found in these organs. KLK6 can hydrolyze the amyloid precursor protein, which is important in the deposition of amyloid plaques and regulation of neural plasticity [62–64]. KLK6 also cleaves myelin basic protein in oligodendrocytes and Schwann cells in the central nervous system in a manner that is related to demyelinating diseases, such as multiple sclerosis [65]. These studies provide evidence that KLK enzymes play a role in diverse patho-physiological processes in human.

PSA/KLK3, a member of KLK gene family, is a well established and clinically-used biomarker for prostate cancer [66]. Recent studies have associated the function of KLKs with the progression of human cancer. Both KLK2 and PSA/KLK3 degrade insulin growth factor binding protein (IGFBP)2, IGFBP3, IGFBP4 and IGFBP 5, thereby releasing the insulin growth factor-1 (IGF1) and regulating the survival and proliferation of both normal and cancerous prostate cells [67, 68]. KLK4 can activate other enzymatic pathways, e.g. MMP [69], pro-uPA to uPAwhich binds to its receptor uPAR [70]. Interestingly, however, uPAR can be cleaved by KLK4 leading to inactivation of this receptor [70]. Hence, KLK4 is an important mediator of the uPA/uPAR axis which is known to have an important role in cancer invasion [71, 72]. A recent study shown that KLK7 cleaves proMMP9 generating an active MMP9 fragement but not by other proteases [69]. More recent studies have demonstrated that several KLKs activate a family of G-protein coupled receptors, PARs. For example, PAR1 is activated by KLK1, KLK4–6 and KLK14, PAR2 by KLK2, KLK4–6 and KLK14, and PAR4 by KLK1 and KLK6, leading to the activation of downstream signaling pathways [73–75]. We and others have shown that cleavage of PAR2 by KLK4 activates the MAPK signaling pathway leading to the growth of prostate and colon cancer cells [76, 77]. KLK7 induces shedding of the cell adhesion protein E-Cadherin, uPAR and ECM protein, vitronectin (VN) break-down promoting an enhanced cell proliferation, migration and invasion in pancreatic cancer cells [78, 79]. Together, these findings suggest important roles of KLKs not only in normal physiological processes but also in disorders such as, cancer.

## Expression of KLKs in ovarian cancer

In the last decades, numerous studies have determined the aberrant expression of members of the KLK family in ovarian cancer [80–82]. Different groups, including ours, have reported that the majority of the KLKs (KLK4–11, KLK13–K15) are aberrantly expressed in ovarian cancerous, compared to normal and benign tissues [12, 80, 81]. It has been reported that at the transcriptional level, KLK4–8, KLK10 and KLK14 are highly expressed in ovarian cancer tissues. Importantly, unbiased gene microarray analyses demonstrated an up-regulated transcription of KLK5–8 and KLK10 in EOC [83, 84]. High protein levels of KLK4–7 and KLK10 have been found in ovarian cancer tissues, while KLK5–8, KLK10, KLK11, KLK13 and KLK14 were found in ascites fluid from ovarian cancer patients as reviewed [81, 85, 86]. Interestingly, KLK6 and KLK10 have shown a strong potential as clinical serum biomarkers for this cancer [81].

Of further clinical relevance, high mRNA and/or protein levels of KLK4–7, KLK10 and KLK15 are associated with shorter progression-free and overall survival time of patients [87–92]. Significantly, the up-regulated expression of KLK4–7, KLK10 and KLK15 is associated with high grade and late stage disease, belonging to the more aggressive Type-II tumors [87–92]. Interestingly, KLK5–7 expression in EOC tissues displayed a closer association with a larger remaining tumor following surgery, higher grade and later stage disease than CA125 [93], the currently used biomarker for women with this cancer. A recent study demonstrated that the expression of KLK6 in stromal cells was associated with aggressiveness of this disease, suggesting its role in the EOC microenvironment [94]. High serum levels of KLK5 [95, 96], KLK6 [97, 98] and KLK10 [99] were associated with poor outcome in women with EOC, suggesting their potential as alternative biomarkers for this malignancy. KLK6–8 and KLK10 have been found to be more specific than KLK4, KLK5, KLK11, KLK14 and KLK15 in EOC [100] in differentiating between benign and other malignant secretions in ascites and pleural effusion fluid. In addition, we and others have reported that high tumor levels of KLK4 and KLK7 were associated with chemoresistance in patients with this cancer [101–103]. On the other hand, levels of KLK8, KLK9, KLK11, KLK13 and KLK14 in tumor tissues were higher in early stage disease, when optimal debulking surgery had been performed, and in those patients who responded to chemotherapy and had a long survival time [91, 104–106]. Our studies, and those from others, showed that KLK4–8 and KLK10 are expressed at high levels in EOC, in particular in high grade serous EOC [52, 89, 107–109]. A strong correlation within the cluster of kallikreins, KLK5–8, KLK10 and KLK11 in EOC tissue samples has been reported [110] suggesting their co-expression in this disease.

Recent studies using genome-wide microRNA (miRNA) profiling approaches have demonstrated the association between expression of miRNAs with pathogenesis, and their potential assistance in diagnosis and prognosis of ovarian cancer. miRNAs have been shown as tumor suppressors or oncogenes, and can also target different cell signaling pathways [111]. For example, let-7a-2 targets KRAS and IL6 functioning as a tumor suppressor, while miR-21 regulates PTEN as an oncogene. Recent bioinformatics analyses have predicted the potential regulation of KLK peptidases by multiple miRNAs [112]. To confirm these findings, White et al. have shown that transfection of let-7f, miR-224, or miR-516a in EOC OVCAR-3 cells reduced expression of KLK10 at both mRNA and protein levels, and cell proliferation [112]. In addition, transfection of let-7f miRNA significantly decreased KLK6 and KLK10 secretion into conditioned media, indicating that one single miRNA targets multiple KLKs [113]. These studies provide evidence that KLK expression is regulated by miRNAs which have a role in post-transcriptional regulation of KLK peptidases.

Together, differential expression of KLKs not only has potential as EOC biomarkers, but may indicate a critical role of these peptidases in the ovarian cancer microenvironment promoting progression and chemo-resistant metastasis, especially in the aggressive Type-II tumors.

## Functions of KLKs in ovarian cancer metastases

### KLKs enhance EOC multicellular aggregation in 3-dimensional (3D) microenvironments

**Fig. 2:**
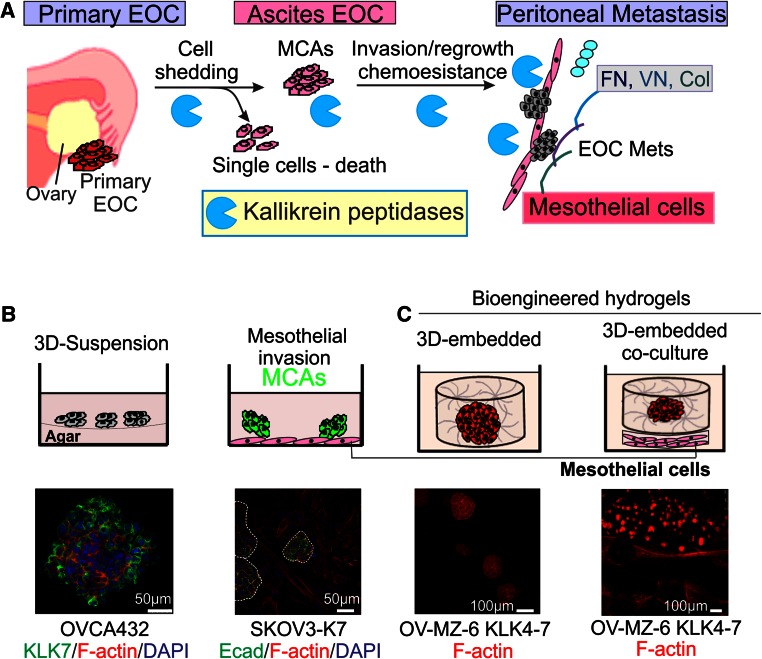
Cell culture platforms to mimic EOC metastasis. **a** Schematic diagram showing potential roles of KLKs in EOC peritoneal dissemination. **b** *Top left panel*, 3D-suspension cultures to mimic the EOC cells growing in the ascites fluid microenvironment. *Top right panel*, MCAs invading into a monolayer of mesothelial cells to mimic peritoneal invasion. *Bottom left panel*, MCAs formed by OVCA432 cells stained with anti-KLK7 and AlexaFluor488 (*green*), F-actin stained with AlexaFluor568 phalloidin (*red*), nuclei stained with 4′,6-diamidino-2-phenylindole (DAPI, *blue*). *Bottom right panel*, invasion into mesothelial cell monolayer by KLK7-expressing SKOV3 cell (SKOV3-K7) MCAs stained with anti-E-Cadherin (Ecad) and AlexaFluor488 (*green*), F-actin stained with AlexaFluor568 phalloidin (*red*), nuclei stained with DAPI (*blue*). **c** *Top panel*, schematic showing ovarian cancer cells embedded and cultured in 3D bioengineered hydrogels, alone (*left*) and sheet (*right*) co-culture with mesothelial cells. *Bottom panel*: KLK4–7-expressing OV-MZ-6 cells grown in the above models. F-actin filaments stained with rhodamine-415 conjugated phalloidin (*red*) and imaged by confocal laser scanning microscopy. *Scale bars* as indicated. With permission from Walter de Gruyter to use the elements of the figure in the published book ‘Kallikrein-related peptidases’ [114]

As KLK4 and KLK7 are up-regulated in EOC cells and tumor tissue samples, with high levels associated with poor outcome in women with this disease [87, 102???], we over-expressed these peptidases in SKOV3 EOC cells to determine their function. We found a reduced migration of SKOV3 cells stably over-expressing KLK4 or KLK7, but no significant changes in cell proliferation in conventional 2-dimensional (2D) monolayer cultures. To investigate the role of these enzymes in the ovarian tumor microenvironment (Fig. 2a) [4, 28], we established a 3-dimensional (3D)-suspension culture (Fig. 2b) to mimic the ascites fluid seen in patients [114]. It is known that homotypic cell adhesion promotes cell survival by forming MCAs in the ascitic fluid suspension microenvironment, and we observed the formation of compact MCAs in KLK4-expressing SKOV3 cells, as did SKOV3 cells treated with recombinant active KLK4 [103]. MCA formation was reduced by treatment with a KLK4 blocking antibody or the selective active-site KLK4 sunflower trypsin inhibitor (SFTI-FCQR), further supporting the role of this peptidase in EOC. KLK4-expressing SKOV3 cells had high levels of the KLK4 substrate, uPA, particularly in 3D-suspension, and high levels of both KLK4 and uPA were observed in patient cells taken from ascites [103]. Lipocalin 2, also named neutrophil gelatinase-associated lipocalin, has been linked to an EOC phenotype [115], and we showed its induction in KLK4-expressing SKOV3 cells and further up-regulation in the 3D-suspension microenvironment [114].

Similar observations were also made in KLK7-expressing SKOV3 cells cultured in 3D-suspension [102] and in the endogenous KLK7 expressing OVCA432 cells (Fig. 2b, left panel). Different from KLK4, however, increased levels of α5/β1 integrins and enhanced adhesion to FN and VN, which was inhibited with a β1 integrin blocking antibody, were observed. Blocking MCA using antibodies against KLK7, α5β1 and β1 integrins confirmed the involvement of KLK7 and integrin-regulated cell adhesion. Importantly, these MCAs invade into a monolayer of peritoneal mesothelial LP9 cells and form cancer cell foci (Fig. 2b, mid panel). We also found that EOC cells derived from ascites fluid expressed higher levels of KLK7 and α5β1 integrin compared to their matched primary tumor cells [102]. Our findings suggest a mechanism for the association of high KLK7 levels with poor prognosis for serous EOC patients by promotion of peritoneal dissemination and invasion via increased MCA formation and α5β1 integrin-mediated cell adhesion. These findings suggest a role of KLKs in EOC metastasis due to the formation of MCA and peritoneal invasion, but the underlying mechanisms differ as KLK4 acts via uPA whilst KLK7 induced integrin-associated pathways.

**Fig. 3:**
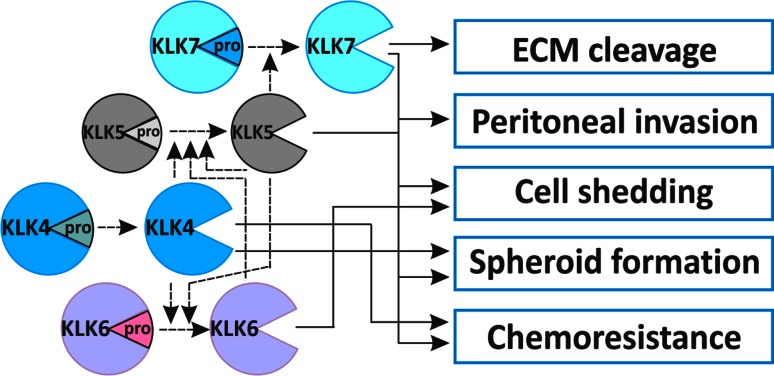
Schematic diagram showing the biochemical activation cascade of KLK4, KLK5, KLK6 and KLK7 (*dashed lines*) and their roles in different aspects of ovarian cancer metastasis (*continuous lines*). *ECM* extracellular matrix

As noted as above, an in vitro biochemical feature of the members in the KLK family is an enzymatic cascade between different KLKs [116, 117]; KLK4 activates proKLK6 and proKLK5, KLK5 auto-activates and activates proKLK6 and proKLK7, KLK6 auto-activates and partially activates proKLK5 (Fig. 3). Importantly, parallel expression of these KLKs has been shown in EOC [12], indicating their interacting roles in this disease (Fig. 3). A previous study demonstrated that simultaneous over-expression of KLK4, KLK5, KLK6 and KLK7 (KLK4-7) in OV-MZ-6 EOC cells significantly increased their invasive behavior in an in vitro transwell Matrigel assay [118]. These KLK4–7 expressing OV-MZ-6 cells increased tumor growth in an animal model compared to OV-MZ-6 cells expressing the single KLK4, KLK5, KLK6 or KLK7, or vector controls. To explore the underlying mechanism, our group has recently reported that KLK4–7 expressing OV-MZ-6 cells had reduced levels of α5β1 and αvβ3 integrins, leading to reduced adhesion to the ECM proteins FN and VN [119].

To understand the crucial mechanism underlying the interaction between EOC cells and their extracellular microenvironment, we have employed a bioengineered hydrogel platform [120] (Fig. 3c). This 3D platform comprises synthetic protease-sensitive hydrogels that are formed from peptide-functionalized polyethylene glycol macromolecules via a factor XIII-catalysed reaction, similar to the fibrinogen cross-linking that occurs during natural fibrin coagulation [121, 122]. Interestingly, over-expression of KLK4–7 did not alter the proliferation rate of OV-MZ-6 EOC cells when cultured as conventional 2D monolayers [118]. However, KLK4–7 expressing OV-MZ-6 cells formed more and larger spheroids when cultured in 3D bioengineered matrices [114, 123]. In particular, a much higher growth rate of KLK4–7 expressing spheroids was seen when they were co-cultured with mesothelial cells, implying their growth-promoting effect on EOC cells [114]. Together, we have demonstrated that KLK peptidases promoted EOC cell survival, MCA formation, adhesion to ECM proteins and invasion into the peritoneal microenvironment, ultimately leading to metastasis.

### Chemoresistance is mediated by KLK proteases in EOC

Chemoresistance is a major obstacle to overcome for prolonging the survival time of women with EOC. A previous study showed that high levels of KLK10 (also known as the normal epithelial cell-specific 1, NES1) in tumor tissue predicted tamoxifen resistance in breast cancer patients [124]. An early study showed that expression of KLK4 in ovarian cancer tissues is associated with resistance to a first line, clinically-used chemo-agent paclitaxel in EOC patients [125]. We have recently confirmed this finding [103] and also demonstrated that high levels of KLK7 in tumor tissues are associated with chemoresistance in EOC patients [102]. It has been documented that MCAs, representing a 3D architecture, are more resistant than 2D monolayer cells [126], and compact MCAs are less responsive to various treatments, including chemotherapies, than scattered and small MCAs [127]. In in vitro studies, we observed that KLK4, but not KLK7-expressing SKOV3 cells are more resistant to cisplatin in 2D monolayer cultures [102, 103]. However, MCAs formed by either KLK4 or KLK7 expressing SKOV3 cells are less responsive to paclitaxel than the vector and native control cells when cultured in 3D-suspension as MCAs. Interestingly, adding the KLK4 selective SFTI–FCQR not only reduced the compaction of KLK4-expressing MCAs but importantly increased their response to paclitaxel to a greater degree than the general serine protease inhibitor, aprotinin [103]. Lipocalin 2, has been associated with chemoresistance [128, 129] and is up-regulated in KLK4 expressing SKOV3 cells cultured as MCAs in 3D suspension [114]. Furthermore, KLK4–7 expressing OV-MZ-6 cells were less responsive to paclitaxel, but not carboplatin, in both 2D monolayers and 3D bioengineered matrices [119]. In search of the underlying mechanism, we reported that the reduced paclitaxel response is independent of MAPK signaling [119]. Interestingly, the levels of α5β1 and αvβ3 integrins decreased in KLK4–7-expressing OV-MZ-6 cells, but adding paclitaxel increased the level of α5 integrin, suggesting its role in paclitaxel resistance induced by these four KLKs [114, 119]. These data imply the potential of inhibition of KLK action as part of a combined therapeutic approach for EOC patients, especially those with high KLK levels in their tumors.

### Proteolytic functions of KLKs in ovarian cancer metastasis

In vitro biochemical studies demonstrated that KLK enzymes function via extracellular hydrolysis or proteolytic activity, such as cleavage of cell–cell adhesion proteins, membrane bound proteins and receptors, cytokines and growth factors, ECM proteins, and proteases including KLKs. For example, KLK4–7 regulate the break-down of the ECM proteins fibronectin (FN), VN, laminin and Type-I and IV collagens [63, 130–134]. All these proteins form part of the ECM that underlies the mesothelial cell layer lining the abdominal cavity which is the most common EOC metastatic site [135, 136]. The cleaved ECM products can contribute to an altered cellular phenotype and cell function, promoting migration and invasion of EOC cells leading to the establishment of metastasis [137–139]. It has been reported that MMP2 cleaves FN and VN into small fragments and increases binding of EOC cells to these fragments and their receptors α5β1 and α3β1 integrins promoting peritoneal adhesion and invasion [136]. It has also been documented that FN is a substrate of KLK7 [134] and we showed that KLK7 over-expression in SKOV3 EOC cells induced their adhesion to this substrate and the level of its receptor α5β1 integrins increased [102]. In addition to the ECM components, proteases can also be substrates of KLK enzymes. KLK4 can cleave uPA, to generate its active form, and also its receptor uPAR [70, 140], both known to be induced in EOC cells in the ascites fluid [141], and associated with chemoresistance and poor prognosis in patients with EOC [142, 143]. KLK7 can cleave the cytokine interleukin (IL)-1β converting it to its active form [144] which can modify the morphology of mesothelial cells, allowing EOC cells to invade into the ECM beneath the peritoneal membrane [145]. KLK6 [146] and KLK7 [78] cleave the cell adhesion protein E-cadherin generating shed fragments which are at a high level in metastatic EOC and ascites [137]. Interestingly, loss of E-cadherin has been associated with EOC progression, parallel to MMP9 [11] which is also a substrate of KLK7 [69]. These data generated from patient samples in clinical studies, along with their biochemical activity, implicate KLKs in the progression and chemoresistance of metastatic EOC.

### Non-proteolytic functions of KLKs in ovarian cancer

Although KLK peptidases function via extracellular hydrolysis to exhibit their enzymatic activity, recent studies have shown their non-proteolytic function in ovarian cancer both in vitro and in vivo. In the KLK gene family, more than 80 splicing variant transcripts have been identified [147, 148]. We have previously reported the simultaneous expression of the wild type transcript encoding the full-length KLK7 and the mRNA variant for an N-terminal truncated KLK7–181 protein in EOC but not in normal ovarian epithelial cells [107]. In later functional studies, we observed that similarly to wild type KLK7, KLK7–181-expressing SKOV3 cells showed increased adhesion to the ECM proteins FN and VN, formed compact MCAs and were more resistant to paclitaxel treatment, suggesting a role of this KLK7–181 variant which is not enzymatically active [102]. We have also found that active KLK4 enzyme treated SKOV3 cells formed compact spheroids that were larger than those treated with the non-active mutant KLK4 (serine to alanine mutation at catalytic triad) which in turn were slightly larger than those seen for vector and native control cells [103]. These data indicate that the action of KLK4 in MCA formation involves both proteolytic and non-proteolytic effects. In addition, KLK10 (NES1) was identified from breast cancer cells but is down-regulated in the progression of EOC [149]. Over-expression of KLK10 in ES2 EOC cells reduced their anchorage-independent growth in vitro and caused a smaller tumor burden in an animal model compared to vector controls [150]. Adding recombinant KLK10 protein into cultures confirmed these findings, supporting its role as a tumor suppressor in EOC progression both in vitro and in vivo. However, the recombinant KLK10 added lacked catalytic activity, indicating a non-proteolytic function in the progression of this disease. These findings are consistent with an earlier study in which two blocking antibodies against PSA/KLK3, one inhibiting the enzymatic activity and the other one not affecting the catalytic activity, both inhibited the biological function of this KLK, suggesting that PSA also has an alternative action via protein–protein interactions [151].

In summary, we have reviewed recent studies on the role of kallikrein peptidases in ovarian cancer progression and resistance to chemotherapy. Importantly, our findings and those of others have provided evidence that both 3D-suspension models and bioengineered matrices can successfully be used as in vitro 3D platforms for research into the functional roles of KLK enzymes on ovarian cancer metastasis and chemoresistance.

## Future directions

For the past decade, our understanding of ovarian cancer progression and chemo-resistant metastasis has been steadily increasing, including our knowledge of the roles of cancer-associated proteases, such as KLKs. To investigate the roles of KLKs in the development, progression, and in particular the chemo-resistant metastasis of ovarian tumors, we have developed in vitro cell culture platforms that closely mimic the in vivo microenvironment. However, we still face challenges to improve these platforms to examine the function of KLK proteases specifically in different EOC microenvironments. In addition, some KLK enzymes have shown potential as pharmaceutical targets while other KLKs may have potential as part of combined therapeutic approaches. Of note, recent studies have shown the potential of synthetic small inhibitors to target individual KLK enzymes at in vitro biochemical and cellular levels [43, 44, 152]. We are still at an early stage testing the anti-metastatic potential of KLK inhibitors using these 3D platforms, and more effort is needed in developing these assays for application to patient derived tumor cells. Furthermore, how interactions in vitro between postulated proteolytic cascades involving different KLK proteases occurs in biological tissues and other related factors and/or contributors occur remains largely unknown. Therefore, we endeavor to identify the biological substrates of the ovarian cancer associated KLK peptidases and their regulated signaling networks in order to identify the underlying molecular mechanisms and molecular targets. Our increasing knowledge will enable further efforts in search of the most effective approaches to target KLK action in the tumor microenvironment, improving the survival time of women with this cancer as well as their quality of life.
